# Introducing a Controlled Outdoor Environment Impacts Positively in Cat Welfare and Owner Concerns: The Use of a New Feline Welfare Assessment Tool

**DOI:** 10.3389/fvets.2020.599284

**Published:** 2021-01-11

**Authors:** Luciana Santos de Assis, Daniel Simon Mills

**Affiliations:** Animal Behaviour Cognition and Welfare Group, School of Life Sciences, University of Lincoln, Lincoln, United Kingdom

**Keywords:** cats (felis catus), indoor, injury, feline, outdoor, wildlife predation, welfare, welfare assessment tool

## Abstract

There is much debate over the pros and cons of allowing cats to roam freely as opposed to keeping them confined indoors. We surveyed owners who implemented a commercial physical containment system to the outdoors to evaluate their characteristics and the apparent impact of this system on cat welfare and owner perceptions. As part of the latter aim, we also developed a new feline welfare assessment tool based on the mathematical relationship between different measures. The survey was circulated to customers over the preceding 2 years of ProtectaPet® between May and June 2019 and gathered 446 responses. Univariate analyses compared changes following installation on factors such as the amount of time the cat spent outside, other cats entering the owner's garden and owners' concerns about their cat outside. Principal component analysis was used to reduce 21 potential indicators of feline welfare into fewer variables. This resulted in 4 subscales, 2 relating to positive welfare and 2 relating to negative welfare. The effects of installation of the containment system and significant predictors of these four welfare subscales were assessed. The majority of respondents lived in an urban environment with a relatively small garden, had multiple cats and a history of feline trauma associated with a road traffic accident. As expected, the time spent outside significantly increased, while the frequency of other cats entering the garden and owner concern about leaving their cats outside significantly decreased. The 4 welfare subscales grouped into positivity, maintenance behaviors, health issues and fearfulness; installation of the system was associated with significant improvements across all of these. Time spent outside after installation had a significant effect on positivity and, to a lesser extent, maintenance behaviors. Overall, installation was associated with positive changes in both owner and cat quality of life, which seem to be particularly associated with an increased sense of security. This suggests that housing cats within a controlled outside environment with physical barriers can provide a practical solution for many of the problems associated with cats being allowed out.

## Introduction

Domestic cats are one of the most popular pets and the population is increasing in many countries [e.g., (1)]; however, there is no consensus on the best way to house them [e.g., (2–6)]. Owned cats have historically been allowed to freely roam in order to allow many natural behaviors associated with good welfare, such as exploration and hunting. In many countries it is culturally accepted that cats are “free to roam” and can leave their owners' properties (7). However, free-roaming cats have increased exposure to several threats to their own welfare such as infectious disease, fighting, theft and the risk of road traffic accidents (RTAs). In the United Kingdom, death from RTAs are ranked as the sixth most important cause of death in cats of all ages and the second for cats under 5 years old (8–11). Additionally, there is growing concern over the impact of domestic cats on the environment through their predation of wildlife. One estimate suggests that in the order of 92 million animals were killed in the UK during 5 months in 1997 (12); with an estimated 1.3–4.0 billion birds and 6.3–22.3 billion mammals killed annually across the USA (13) and an average of 186 reptiles, birds and mammals killed per year per roaming pet cat has been reported in Australia (14). Although farm/barn cats, strays, cats in supported colonies and feral cats are believed to play a large role in these mortality figures, owned pets are also important contributors since their densities are much higher in residential areas and may be responsible for killing between 4,440 to 8,100 animals per square kilometer per year in an area where they live in Australia (13, 14). Given these figures, the “Cat wars” that rage between ecologists and cat enthusiasts are not surprising [e.g., (6, 15)].

Being kept exclusively indoors is also not without risks to feline welfare [for review see (3)], and there is growing evidence of environmental contamination from home furnishings and dust affecting cat health (16, 17). Keeping cats indoors can cause frustration and unwanted behavioral challenges leading to stress and compromised health, especially in multi-cat homes (4, 18). The decision balance between both owners' beliefs about the relative importance of feline autonomous control and concerns over the impact of restrictions due to containment on a cat's quality of life alongside the perceived potential harms vs. benefits of cats in the community are clearly critical to determining what owners decide to do. Indeed it has been shown that cat owners who never, or only sometimes, contained their cats are more likely to believe that cats have strong physical and emotional needs to be outdoors, and are less confident in their ability to effectively contain their cats than owners who restrict their cats more (2). One potential solution is to allow cats controlled outdoor access through a property-based containment system, such as a cat-proof fence. However, there is a lack of research on the impact of these devices on cat welfare and owner perceptions of well-being.

Therefore, we surveyed owners to assess (1) the profile of owners choosing to contain their cat with some form of containment system (i.e., ProtectaPet^®^ cat fencing solutions) and (2) the apparent impact of this system on cat welfare and owner perceptions of this. As part of the latter aim, we also examined the way in which different potential welfare measures were related to each other in order to produce a feline welfare assessment tool, for future use in similar studies that would allow evaluation in other contexts including with other systems.

## Methodology

### Ethical Approval

This study was approved by the University of Lincoln Research Ethics Committee (ref: 2020-3442).

### Questionnaire

An English language questionnaire was developed in Qualtrics® containing 36 questions about cat and owner demographics, changes in how cats were kept before and after the installation of the containment system and owner opinions regarding importance of allowing their cats access to outdoors vs. concerns. Table 1 shows a summary of these questions whilst the whole questionnaire is available in the Supplementary Material.

**Table 1:** Summary of variables considered in the questionnaire comparing cats' behaviors and owner's concerns ***before***and ***after***the installation of one type of the ProtectaPet^®^ systems.

**Section**	**Number of questions**	**Total number of items**
Owner demographic	9	9 (1 each)
Cat demographic	7	7 (1 each)
Owners' opinion about what they thought was important for the welfare of indoor cats	1	12
Potential value to a cat of having access to the outside	1	4
Attitudinal statements concerning owners' worries about cats being outside before and then after the installation	5	36 (10 + 1 + 10 + 9 + 6)
Comparing the cat's behavior and health before vs. after installation of the physical containment system	1	21
Time spent outside before and after installation, subjective opinion about whether their cats were spending more time outside after the installation,	3	3 (1 each)
Frequency with which other cats visited the owner's garden before and after installation	2	2 (1 each)
Whether the cat had free access to the outside (and if not, which ways were used to contain their cats)	2	2 (1 each)
Whether the owners had experience of a cat injured on the road (and, if so, whether this was fatal)	2	2 (1 each)
Decision process associated with acquiring the system and the most valuable benefit of it	3	3 (1 each)

The survey was circulated to previous customers of ProtectaPet® over the preceding 2 years and made available online between May and June 2019 (~1,800 individuals).

### Subjects

Participants needed to be cat owners, aged 18 years old or over, and have purchased one of the three containment systems for cats provided by ProtectaPet®, i.e., fence barrier, enclosure or catio (see: https://protectapet.com/shop/ for details). There was no other restriction applied. Data were collected anonymously, and participants provided informed consent at the outset (Supplementary Material).

### Data Analysis

#### Preparing the Data

Participants who had not purchased one of the three ProtectaPet® types of system were deleted; incomplete questionnaires were kept where data could be usefully used; no data imputation was undertaken. Accordingly, the total number of questionnaires varied depending on the specific analysis.

Some items were re-coded in order to generate an ordinal sequence, others were collapsed so a new variable comparing ***before***and ***after***installation, i.e., “change in time spent outside” and “change in frequency of other cats visiting the garden.” The new variables indicated whether time or frequency was less, no different or more than before and by how many categorical units to give an ordinal value to any difference.

#### Statistical Analysis

Data analysis was performed using R 3.5.1. (19). Since data were non-parametric, Wilcoxon signed-rank tests were used to initially compare ***before***and ***after***installation of the controlled environment system on simple dependent variables: the time cats spent outside, frequency of other cats visiting your garden, and 10 statements regarding the owners' concern about their cats being outside. Kruskal-Wallis, Chi-square and Fisher's exact tests were used to compare cat and owner features according to the types of system, i.e., fence barrier, enclosure or catio [functions wilcox.test, kruskal.test followed by pairwise.wilcox.test with Holm correction, chisq.test and fisher.test, package “stats” – (20)]. In accordance with the recommendations of Perneger (21) as this was an exploratory study no statistical correction was made for multiple testing since the risk of identifying spurious relationships was outweighed by the risk of failing to identify potentially important relationships for future study.

##### Development of the Feline Welfare Assessment Tool

In order to evaluate how the controlled outdoor environment impacted cat welfare, the section of the survey which asked owners about change in 21 behavioral and health elements after installing the system was used. Although the change in each could be used as a dependent variable, we wanted to create meaningful groupings of variables in order to produce a welfare assessment instrument, requiring fewer tests. We used Principal Component Analysis [PCA – package “psych,” function “principal” – (22)] with an oblique rotation (since there was no reason to assume that the constituent behaviors were independent) to identify components to make up subscales. A parallel analysis [package “nFactors,” function “parallel” – (23)] was used to help determine the number of components to extract, alongside visual inspection of the value of all reasonable options and Cronbach's alpha (package “psych,” function “alpha”) was performed to evaluate the internal consistency within each principal component (22). A welfare subscale was then created using the items loading > 0.4 on each of the principal components retained. Each item making up the subscale was scored as either an “empty cell,” “−1,” “0,” or “+1” and multiplied by its loading within a given welfare component. All relevant items were then summed and divided by the total possible score for specific PCs to generate a score with a standardized range between −1 to +1. For example, the score of PC2, consisted of 3 behaviors with loadings of 0.88, 0.85, and 0.75, so a score was calculated from: [(x^*^0.88) + (y^*^0.85) + (z^*^0.72)]/(0.88 + 0.85 + 0.72), where x, y, and z represent the change value (−1, 0 or +1).

##### Predictors of Each Welfare Component Sub-score

To test if there was a change in welfare within the population associated with installation of the system, Wilcoxon signed rank tests were used to evaluate whether the before vs. after comparison for the welfare sub-scores differed from zero.

The source of any potential difference in welfare sub-score was then initially investigated by using separate Kruskal-Wallis tests on each the following independent variables: the type of system installed (i.e., Fence barrier, Enclosure and Catio); whether the cat used to have unsupervised access to the outside before installation; what type of access to outside the cat used to have before installing (i.e., no access at all, controlled access or no restriction). General linear models [package “stats,” function “lm” – (20)] were used to determine which of a range of factors significantly affected each welfare sub-score using a stepwise method, i.e., all variables were initially included in the models and were serially removed to produce minimal adequate models [based on Akaike Information Criterion – AIC; package “MASS,” function “stepAIC,” backward elimination – (24, 25)]. Residuals were visually investigated and, after removing outliers (nine cats each for Health issues, Positivity and Fearfulness, and six for Maintenance behaviors sub-scores), they showed an approximately normal distribution. The independent variables included in the full model were: the type of system installed; the owner gender; the owner age; the area where they lived; the land around the property; the number of cats in the household; the source of acquisition; if the cat was purebred; the cat gender and whether it was neutered; if the cat was suffering from an ongoing health problem; whether the cat used to have unsupervised access to the outside before installation; whether the cat used to have “no access at all,” “supervised/restricted access,” or “no restriction” to the outside before installation; how long the cat used to stay outside *before* installation; how long the cat stayed outside *after* installation; how often other cats used to enter the garden *before* installation; how often other cats currently enter the garden *after* installation.

## Results

### Respondent Characteristics

Data were collected from 446 questionnaires of which 442 specified which of the three types of system of controlled environment for cats provided by ProtectaPet® they had purchased. One was blank beyond indicating the system purchased, another was a duplicate and one owner answered twice about different cats, thus the full dataset was 443 owners and 444 cats, but there was some missing data for various items.

As can be seen in Table 2, over half of cats were mixed breed (56.9%) and males (54.4%). The majority was neutered (97.5%), vaccinated in the last year (86.4%), microchipped (94.5%) and did not have any significant health problem (86.9%). The main sources of acquisition were “shelter/rescue center” and “purchased from breeder” (37.4 and 30.8%, respectively). Before installing the system approximately half of cats (53.7%) did not have any level of *unsupervised* access to the outside. The level of outside access of 382 cats before installation was as follows: 47.6% had restricted and/or supervised outside access, 30.9% did not have any outside access and 21.5% were allowed unrestricted access. The main method used for containment at this time was supervised access 36.4%.

**Table 2:** Demographics of *cats* whose owners decided to install one type of the ProtectaPet^®^ systems to provide controlled access to the outdoors.

**Variables**		**Number/Total number of answers**	**%**
Gender	Male	237/435	54.5%
	Female		
Neutered	Yes	424/435	97.5%
Breed	Purebred	158/436	36.2%
	Mixed breed	248/436	56.9%
	Unsure	30/436	6.9%
Vaccinated in the last year	Yes	376/435	86.4%
Microchipped	Yes	411/435	94.5%
Presence of a significant health problem	Yes	51/435	11.7%
	No	379/435	86.9%
	Unsure	6/435	1.4%
Source of acquisition	Shelter/rescue center	163/436	37.4%
	Purchased from breeder	134/436	30.8%
	Given by friend/familiar	44/436	10.1%
	Stray	35/436	8%
	Advertisement online/newspaper	17/436	3.9%
	Inherited from previous owner	8/436	1.8%
	Pet shop	1/436	0.2%
	Other: other form of rescue or were home bred	34/436	7.8%
Unsupervised access to the outside at least some of the time *before* installing the system	Yes	185/430	43%
	No	231/430	53.7%
	Adopted only after installation	14/430	3.3%
Level of outside access *before* installing the system	Restricted and/or supervised outside access	182/382	47.6%
	Did not have any outside access	118/382	30.9%
	Unrestricted access	82	21.5%
Main method(s) used for containment *before* installing the system	Supervised access	96/264	36.4%
	High fence/wall	45/264	17%
	Other cat enclosure	36/264	13.6%
	Electronic flap	19/276	7.2%
	Others: cat harness and curfew during night	51/267	19.3%
	No specific method in place	82/264	31.1%

Regarding the owners (Table 3), most respondents identified as female, and the median age was in the 46 to 55 years old category. Half of respondents (50.6%) lived in an urban environment with 65% describing the land around their property as a medium sized garden. The majority of owners (81.9%) owned more than 1 cat, with a median of 2 cats per house. Most owners (56.4%) had had a cat injured on the road and for 82.4% the injury had been fatal.

**Table 3:** Demographics of *cat owners* who decided to install one type of the ProtectaPet^®^ systems to provide controlled access to the outdoors.

**Variable**		**Number/Total number of answers**	**%**
Gender	Female	347/442	78.5%
	Male	91/442	20.6%
	Other or preferred not to say	4/442	0.9%
Age categories	18 to 25 years old	1/442	0.2%
	26 to 35 years old	47/442	10.6%
	36 to 45 years old	77/442	17.4%
	46 to 55 years old	136/442	30.8%
	56 to 65 years old	109/442	24.7%
	66 years old or over	72/442	16.3%
Area where they live	Urban environment	221/437	50.6%
	Semi-rural environment	177/437	40.5%
	Rural environment	39/437	8.9%
Land around their	Small garden/yard	127/437	29.1%
property	Medium sized garden	284/437	65%
	Substantial grounds	26/437	5.9%
Number of cats	1 cat	79/437	18.1%
owned	2 cats	174/437	39.8%
	3 cats	76/437	17.4%
	4 cats	42/437	9.6%
	5 or more cats	66/437	15.1%
Owners who had a cat injured on the road	Yes	216/383	56.4%
Cats who died from this injury	Yes	178/216	82.4%

The attitude of owners to the series of questions relating to impact and management of cats going outdoors are summarized in Table 4. It shows that the majority of owners (77.0%) either agree or strongly agree that cats have a better quality of life when having access to outdoors, whilst very few agree that the government should implement curfews for cats or that wandering cats are a nuisance (7.1 and 16.2%, respectively).

**Table 4:** Strength of feeling of 378 cat owners concerning the impact and management of cats going outdoors.

**Question**	**Strongly disagree**	**Disagree**	**Neither agree nor disagree**	**Agree**	**Strongly agree**
A cat that has access to the outdoors has a better quality of life than one that does not.	7 (1.8%)	25 (6.6%)	55 (14.5%)	127 (33.6%)	164 (43.4%)
Cat owners should all have a specific method for containing their cats	8 (2.1%)	58 (15.3%)	161 (42.6%)	95 (25.1%)	56 (14.8%)
It is the owners responsibility to keep their cat from wandering	13 (3.4%)	58 (15.3%)	122 (32.3%)	126 (33.3%)	59 (15.6%)
Owners are responsible for any problems their cat can cause their neighbors	12 (3.2%)	38 (10.0%)	83 (22.0%)	182 (48.1%)	63 (16.7%)
The government should implement curfews for cats	175 (46.3%)	103 (27.2%)	73 (19.3%)	16 (4.2%)	11 (2.9%)
Wandering cats are a nuisance	86 (22.7%)	114 (30.2%)	117 (30.9%)	49 (13.0%)	12 (3.2%)

Access to the outside was viewed by owners as beneficial in a number of ways such as for exercise, to explore/general enrichment and express natural behavior (Table 5). The main requirements deemed very important to indoor cats (>90%) were litter tray, scratching post, food, toys and vantage points (Table 6). Many of them could easily be met by outdoor access.

**Table 5:** Opinion of 383 cat owners regarding the importance of outside access to their cats.

**Advantage from outside access**	**Not a benefit**	**Slightly beneficial**	**Somewhat beneficial**	**Very beneficial**	**Extremely beneficial**
Exercise	1 (0.3%)	8 (2.1%)	32 (8.4%)	105 (27.4%)	237 (61.9%)
Opportunities for hunting	85 (22.2%)	80 (20.9%)	107 (27.9%)	65 (17.0%)	46 (12.0%)
Increased space to wander explore/general enrichment	3 (0.8%)	8 (2.1%)	26 (6.8%)	93 (24.3%)	253 (66.0%)
Increased freedom to express natural behavior	2 (0.5%)	5 (1.3%)	33 (8.6%)	93 (24.3%)	250 (65.3%)

**Table 6:** Opinion of 429 cat owners regarding the items they think are very important to provide to an indoor-only cat.

**Item**	**Count**	**%**
Litter tray	418	97.4%
Scratching post	410	95.6%
Food	409	95.3%
Toys	397	92.5%
Vantage points / windows to look out from	388	90.4%
Places to hide	376	87.6%
Companionship	376	87.6%
Bed	360	83.9%
Access to fresh air, e.g., slightly opened window	351	81.8%
Specific access to a sunny spot	344	80.2%
Specific devices to encourage exercise (but not play)	289	67.4%
Forms of enrichment not listed, please give brief details (main ones: Interactive/stimulating toys/food, Interaction/play with owner, Catnip/grass, Providing higher play areas, Water)	112	26.1%

### Changes *Before* vs. *After* Installation of the ProtectaPet® System

#### Overall

**Figure 1:**
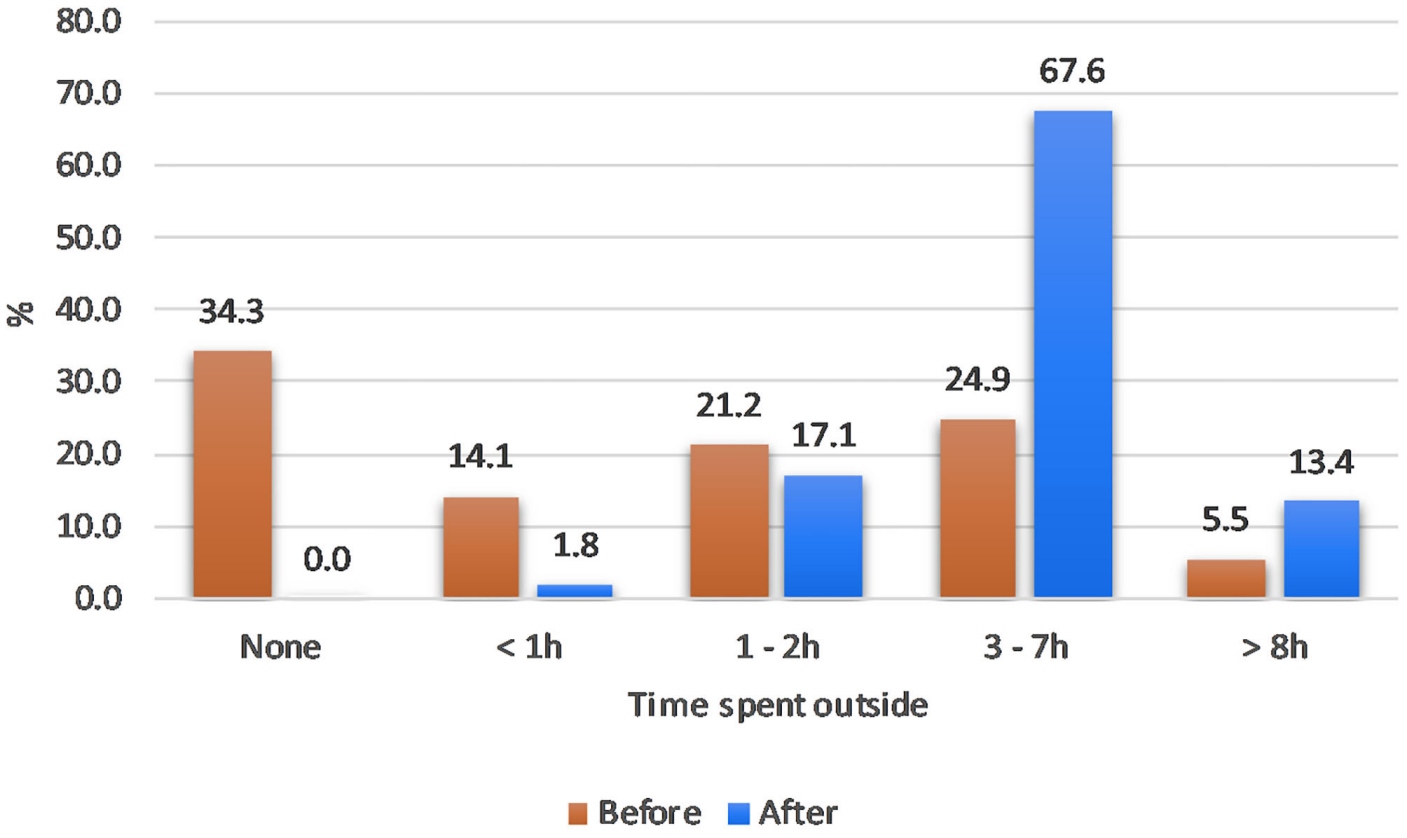
Percentage of cats that spent time outside according to each time interval *before* vs. *after* the installation of one of the ProtectaPet® systems for controlled environment.

Cats were spending significantly more time outside after installation of the containment system (Figure 1, “Hours spent outside”: V = 1,953, *p* < 0.001, ***Before*** median = 1–2 h, ***After***median = 3–7 h). Before installation, 131/382 (34.3%) cats did not have any access to outside, 54/382 (14%) had less than an hour's access, 81/382 (21.1%) from 1 to 2 h, 95/382 (24.9%) from 3 to 7 h, and 21/382 (5.5%) more than 8 h. By contrast, after installing the system, all cats had some outdoor access and over 98% more than 1 h (65/380, 17.1% 1–2 h; 257/380, 67.6% 3–7 h; and 51/380, 13.4% more than 8 h). Of those who provided a response, 299/382 (78.3%) owners indicated that their cat had greater outdoor access now that the system had been installed.

The perceived frequency of visits of other cats significantly decreased after installation of the system (V = 49,854, *p* < 0.001, ***Before***median = more than once a week, ***After***median = less than once a month). Likewise, the owners' level of concern about 10 possible problems associated with their cats having unrestricted access to the outside were significantly reduced (all *p*-values < 0.001 – Table 7). The safety provided by the system was the leading reason for investing in it, with 288/379 (76%) rating it among the two most important factors that influenced their selection of the type of ProtectaPet® system.

**Table 7:** Number of cat owners who reported being “Very concerned” or “Extremely concerned” about potential issues relating to their cats going outside *before* and *after* installing the ProtectaPet^®^ system (*n* = 380).

**Question**	**Before installation**		**After installation**	
	**Very concerned**	**Extremely concerned**	**Very concerned**	**Extremely concerned**
Injury on the road	70 (18.4%)	253 (66.6%)	2 (0.5%)	2 (0.5%)
Death on the road	65 (17.1%)	266 (70.0%)	2 (0.5%)	3 (0.8%)
Getting lost	91 (23.9%)	177 (46.6%)	4 (1.0%)	1 (0.3%)
Killing wildlife	47 (12.4%)	46 (12.1%)	10 (2.6%)	2 (0.5%)
Conflict with other cats	93 (24.5%)	109 (28.7%)	3 (0.8%)	2 (0.5%)
Conflict with other animals	77 (20.3%)	101 (26.6%)	2 (0.5%)	1 (0.3%)
Problems for neighbors	43 (11.3%)	57 (15.0%)	0 (0%)	1 (0.3%)
Poisoning	80 (21.0%)	140 (36.8%)	2 (0.5%)	2 (0.5%)
Theft	66 (17.4%)	151 (39.7%)	3 (0.8%)	3 (0.8%)
Getting trapped	84 (22.1%)	144 (37.9%)	4 (1.0%)	1 (0.3%)

Many health and behavioral potential indicators of stress appeared to show improvement as a result of installation of the system (Table 8).

**Table 8:** Changes in health and behavior of cats as reported by their owners (*n* = 382) *after* installing a ProtectaPet^®^ system. Owners were asked to report whether the behavior changed from *before* the installation.

**Behaviors**	**Condition has never occurred in my cat**	**Less severe/frequent now**	**No change**	**More severe/frequent now**
Physical injuries – bites/scratches	253 (66.2%)	47 (12.3%)	79 (20.7%)	3 (0.8%)
Physical injuries – more serious	281 (73.6%)	33 (8.6%)	65 (17.0%)	3 (0.8%)
Unexplained changes in mood	218 (57.1%)	46 (12.0%)	110 (28.8%)	8 (2.1%)
Soiling in the home with either urine or feces, including spray marking	258 (67.5%)	39 (10.2%)	67 (17.5%)	18 (4.7%)
Cystitis/bladder problems diagnosed by the vet	299 (78.3%)	18 (4.7%)	17 (65.0%)	0 (0.0%)
Respiratory/breathing problems	316 (82.7%)	4 (1.0%)	58 (15.2%)	4 (1.0%)
Skin allergies/persistent scratching	292 (76.4%)	14 (3.7%)	71 (18.6%)	5 (1.3%)
Hairballs	129 (33.8%)	38 (9.9%)	210 (55.0%)	5 (1.3%)
Disturbance at night – crying/running around/waking you up	173 (45.3%)	62 (16.2%)	135 (35.3%)	12 (3.1%)
Willingness to play with you	29 (7.6%)	27 (7.1%)	270 (70.7%)	56 (14.7%)
General presence around you	27 (7.1%)	19 (5.0%)	270 (7078%)	66 (17.3%)
Anxiousness/ jumpiness/looking out the whole time	125 (32.7%)	74 (19.4%)	168 (44.0%)	31 (5.9%)
Irritability/aggressive behavior	214 (56.0%)	44 (11.5%)	116 (30.4%)	8 (2.1%)
Hunting behavior/bringing prey home	132 (34.5%)	61 (16.0%)	131 (34.3%)	58 (15.2%)
Thirst	62 (16.2%)	6 (1.6%)	291 (76.2%)	23 (6.0%)
Appetite	37 (9.7%)	18 (4.7%)	300 (78.5%)	27 (7.1%)
Sleeping	32 (8.4%)	18 (4.7%)	295 (77.2%)	37 (9.7%)
Active but relaxed around the home	24 (6.3%)	18 (4.7%)	268 (70.2%)	72 (18.8%)
Hiding away	147 (38.5%)	44 (11.5%)	184 (48.2%)	7 (1.8%)
Clinginess, including excessive meowing	134 (35.1%)	33 (8.6%)	190 (49.7%)	25 (6.5%)
Other, please specify	141 (36.9%)	22 (5.8%)	190 (49.7%)	29 (7.6%)

The main features specified by owners in “Other” were anxiety from seeing other cats outside and aggression with other cats in the household.

#### Differences Between Systems

There were significant differences between the containment systems in relation to “having greater access to outside now” according to their owners. Cats with a full “Cat enclosure” were most likely to see an increase in access to outside followed by those with a “Catio” followed by “Cat fence barrier” (Fisher's exact test, *p* = 0.036; Table 9). However, when analyzing how long the cats spent outside ***before***and ***after***separately, no significant differences were found between the three types of systems.

**Table 9:** Number and percentage of cats that have greater access to outside (according to their owners) after their owners installed one of the three types of ProtectaPet^®^ system.

**Type of ProtectaPet (R) system**	**Greater access to outside**		
	**Yes**	**No**	**Total**
Cat fence barrier	248 (76.3%)	77 (23.7%)	325
Cat enclosure	38 (92.7%)	3 (7.3%)	41
Catio	11 (84.6%)	2 (15.4%)	13

There was no significant difference between the groups based on the system they purchased regarding how often other cats entered the garden before that system was installed (Fence and Enclosure medians = 4: More than once a week, and Catio median = 5: Once a day, respectively). Whereas, ***after***installing a system, those with a Cat fence barrier saw greatest reduction in access by other cats to their garden than those with “Cat enclosure” and “Catio” (medians = 0: Never, 1: Less than once a month, and 2: Once a month, respectively; Kruskal-Wallis X2 = 25.446, df = 2, *p* < 0.001). *Post-hoc* analysis showed that the “Cat fence barrier” provided greater limitation than either “Cat enclosure” or “Catio” (*p* < 0.001), but there was no significant difference between these latter two enclosure types.

Regarding the level of concern expressed by owners ***before***installation, there were no significant differences between the types of system installed. However, ***after***installation owners who installed “Cat fence barrier” were more concerned than “Cat enclosure” (*p* = 0.029) about their cats “getting lost” (Kruskal-Wallis X2 = 6.8701, df = 2, *p* = 0.032); there was a similar trend regarding “injury on the road” and “death on the road” (Kruskal-Wallis X^2^ = 6.3049, df = 2, *p* = 0.043; and Kruskal-Wallis X^2^ = 6.1402, df = 2, *p* = 0.046, respectively) where *post-hoc* analysis did not differ significantly (*p* = 0.081 and 0.087, respectively). Likewise, owners who purchased “Cat fence barrier” showed only a trend to be more concerned about their cats “killing wildlife” than those who installed “Catio” (Kruskal-Wallis X^2^ = 8.3545, df = 2, *p* = 0.015; *post-hoc*: Cat fence barrier > Catio *p* = 0.057).

### Welfare Scores

PCA indicated that up to five principal components (PCs) might be acceptable (Supplementary Material). However, the 5 PC solution had no variable with a loading of >0.4 on the fifth principal component and it added little to the total variance explained, thus a 4-factor solution was selected. Nineteen of the 21 measures were retained since they loaded >0.4 on at least one PC. “Hairballs” and “Disturbance at night – crying /running around/waking you up,” did not load on any PC and so were not included in the welfare sub-scales. Thus, the feline welfare assessment tool (Table 10) consisted of four principal components which explained 57% of total variance. The interpretation given to each PC based on their constituent items was as follows: PC1: “Health issues,” PC2: “Positivity,” PC3: “Maintenance behaviors” and PC4: “Fearfulness.” This terminology will be used for the component sub-scales from here-on, for clarity. Two pairs of factors had correlations over the 32% which indicates commonality and appropriate use of the oblique rotation (26): Health issues and Fearfulness (33%) indicative of poorer welfare; Positivity and Maintenance behaviors (40%) indicative of positive welfare. All PCs showed Cronbach's alpha of at least 0.7 which is considered a good internal consistency between their items (Table 10).

**Table 10:** Results of the principal component analysis performed with 21 measures comparing before and after installation of a ProtectaPet^®^ system in order to provide controlled access to environment in 382 cats.

**Behaviors**	**PC1** **Health issues**	**PC2** **Positivity**	**PC3** **Maintenance behaviors**	**PC4** **Fearfulness**
Physical injuries (more serious)	**0.81**	−0.01	−0.04	−0.03
Respiratory/breathing problems	**0.8**	−0.06	0.07	−0.05
Physical injuries/bites	**0.77**	0.08	0.01	−0.02
Cystitis/bladder problems diagnosed by the vet	**0.76**	0.04	−0.09	0.06
Skin allergies/persistent scratching	**0.71**	−0.02	0.02	0.02
Soiling in the home with either urine or feces	**0.68**	0.07	−0.05	0.04
Unexplained changes in mood	**0.62**	0.04	0.14	0.04
Willingness to play with you	0.04	**0.88**	−0.05	0
General presence around you	0.03	**0.85**	0.01	0.03
Active but relaxed around the home	0	**0.72**	0.17	−0.02
Thirst	−0.01	0.03	**0.77**	0.12
Appetite	−0.06	0.18	**0.77**	0
Hunting behavior/bringing prey home	0.19	−0.26	**0.65**	−0.05
Sleeping	−0.04	0.38	**0.48**	0.03
Clinginess, including excessive meowing	−0.07	0.02	0	**0.83**
Hiding away	0.02	−0.04	−0.01	**0.75**
Others (anxiety from seeing other cats outside and aggression with other cats in the household)	0.06	−0.03	0.09	**0.52**
Anxiousness/jumpiness/looking out the whole time	0.19	0.08	0.16	**0.44**
Irritability/aggressive behavior	0.4	0.01	0.02	**0.43**
Eigenvalues	5.55	2.93	1.28	1.06
Proportion Variance	0.22	0.13	0.11	0.11
Cumulative Variance	0.22	0.35	0.46	0.57
Cronbach's alpha	0.87	0.82	0.7	0.7

*Bold numbers indicate the highest loadings of each behavior and to which principal component they are assigned*.

About half of the population were reported to show changes in each welfare sub-score (i.e., had their behaviors less or more frequent after the installation whilst the other half did not show any change in these behaviors), and each component showed a significant change pre- vs. post- installation; with Health issues and Fearfulness being lower (i.e., less frequent/severe), whilst Positivity and Maintenance behaviors increased (i.e., more frequent/severe; *p* < 0.001, *p* < 0.001, *p* < 0.001, and *p* = 0.015, respectively).

From the univariate analysis, there were no statistically significant differences identified between the types of systems in the welfare sub-scores, but the effects on both Positivity and Maintenance behaviors showed a tendency (Table 11).

**Table 11:** Results of univariate analysis of each welfare sub-score when comparing types of containment system installed and types of access to the outside before installation of a ProtectaPet^®^ system in cats.

**Variables**	**Welfare sub-score**	**Kruskal-Wallis or Mann-Whitney**	***Post-hoc* test (Holm correction)** **or direction**
Types of systems	Health issues	H = 0.86352, df = 2, *p* = 0.6494	
	Positivity	H = 4.839, df = 2, *p* = 0.08897	Catio < Fence (*p* = 0.082) Catio < Enclosure (*p* = 0.1)
	Maintenance behaviors	H = 5.0868, df = 2, *p* = 0.0786	Catio < Fence (*p* = 0.071) Catio < Enclosure (*p* = 0.071)
	Fearfulness	H = 1.2737, df = 2, *p* = 0.529	
Level of restricted access to the outside ***before*** installing the system	Health issues	H = 9.4073, df = 2, *p* = 0.009	“Supervised/restricted access” < “No access” (*p* = 0.016) “Unrestricted access” < “No access” (*p* = 0.011)
	Positivity	H = 1.3894, df = 2, *p* = 0.499	
	Maintenance behaviors	H = 12.414, df = 2, *p* = 0.002	“Supervised/restricted access” < “No access” (*p* = 0.095) “Unrestricted access” < “No access” (*p* = 0.0018) “Unrestricted access” < “Supervised/restricted access” (*p* = 0.03)
	Fearfulness	H = 3.5407, df = 2, *p* = 0.17	
Unsupervised access to outside ***before*** the installation (Yes vs. No)	Health issues	W = 4790.5, *p* = 0.0005	“Unsupervised” < “Not unsupervised”
	Positivity	W = 7222, *p* = 0.08	
	Maintenance behaviors	W = 3971.5, *p* = 9.957e-08	“Unsupervised” < “Not unsupervised”
	Fearfulness	W = 7132, *p* = 0.1363	

When comparing welfare sub-scores on the basis of the level of restricted access to the outside which cats had before installing the system, significant differences were found for Health issues and Maintenance behaviors. Cats that did not have any access to outside showed higher Health issues scores than ones who used to have either supervised/restricted or unrestricted access to outside (*p* = 0.016 and 0.011, respectively). Similarly, cats who did not have either any access or supervised/restricted access to outside scored higher for Maintenance behaviors than those with unrestricted access to outside (*p* = 0.0018 and 0.03, respectively). Comparing the component sub-scores of cats who used to have unsupervised access to those without this, Health issues and Maintenance behaviors also showed significant differences where cats with “Not unsupervised” scored higher for both sub-scores than cats that had “Unsupervised” access to the outside (Table 11).

#### Possible Predictors for Each Welfare Sub-score

The minimal adequate models (MAM) resulting from the GLM relating to each welfare component are described below and in Table 12.

**Table 12:** Minimal adequate model (MAM) for each welfare component with estimated coefficient (β), standard error, *t*-value, and *p*-value.

	**β**	**Std. Error**	***t*-value**	***p*-value**
**Health issues sub-score**				
(Intercept)	−0.167	0.048	−3.467	0.006
Owner age	0.019	0.008	2.553	0.011
Area where they live (urban vs. semi-rural)	−0.042	0.019	−2.221	0.027
(urban vs. rural)	−0.039	0.033	−1.197	0.233
Land around property (small vs. medium-sized garden)	0.033	0.019	1.719	0.087
(small vs. substantial garden)	−0.012	0.041	−0.294	0.769
If the cat was purebred (Yes vs. No)	−0.053	0.020	−2.705	0.007
(Yes vs. I do not know)	0.001	0.036	0.035	0.972
Unsupervised access to outside *before* the installation (Yes vs. No)	0.049	0.018	2.773	0.006
*Adjusted R-squared: 10.05%*				
**Positivity sub-score**				
(Intercept)	0.028	0.184	0.150	0.881
Owner age	−0.040	0.020	−2.018	0.045
If the cat was inherited from a previous owner (No vs. Yes)	0.569	0.172	3.299	0.001
Unsupervised access to outside ***before*** the installation (Yes vs. No)	−0.086	0.047	−1.846	0.066
Time spent outside ***after*** the installation	0.086	0.035	2.432	0.016
*Adjusted R-squared: 8.76%*				
**Maintenance behaviors sub-score**				
(Intercept)	0.061	0.102	0.598	0.550
Owner age	−0.024	0.010	−2.359	0.019
Area where they live (urban vs. semi-rural)	−0.040	0.026	−1.506	0.133
(urban vs. rural)	0.038	0.044	0.872	0.384
Land around property (small vs. medium-sized garden)	0.050	0.028	1.765	0.079
(small vs. substantial garden)	0.145	0.053	2.686	0.008
Number of cats in the household	−0.017	0.009	−1.911	0.057
If the cat was neutered (Yes vs. No)	0.155	0.070	2.215	0.028
(Yes vs. I do not know)	0.247	0.175	1.410	0.160
Unsupervised access to outside ***before*** the installation (Yes vs. No)	0.125	0.024	5.150	6.08E-07
Time spent outside ***after*** installation	0.028	0.019	1.488	0.138
How often other cats entered in the garden ***before*** installation	−0.018	0.007	−2.690	0.008
How often other cats currently enter in the garden ***after*** installation	−0.018	0.008	−2.202	0.029
*Adjusted R-squared: 22.32%*				
**Fearfulness sub-score**				
(Intercept)	−0.207	0.058	−3.541	0.000
Land around property (small vs. medium-sized garden)	0.053	0.030	1.777	0.077
(small vs. substantial garden)	−0.156	0.060	−2.596	0.010
Number of cats in the household	−0.022	0.010	−2.179	0.030
If the cat was purebred (Yes vs. No)	0.094	0.030	3.102	0.002
(Yes vs. I do not know)	0.164	0.059	2.794	0.006
If the cat had ongoing significant health problem (Yes vs. No)	0.067	0.038	1.786	0.076
Time spent outside ***before*** the installation	0.021	0.010	2.034	0.043
*Adjusted R-squared: 14.19%*				

The adjusted R-squared indicates the total proportion of variance explained by each model. For nominal variables each row shows the contrast between the reference category first vs. the available alternatives (second). For ordinal variables the relationship described is related to an increase in the variable.

For the Health issues sub-score, the MAM included five variables although land around the property showed only marginal statistical significance as a factor (variables where *p* > 0.05 may be retained, given the use of AIC for model building). Cats with older owners had higher Health issues sub-scores (poorer welfare) possibly along with those with medium rather than small sized gardens; whereas those who lived in semi-rural rather than urban areas, were **not** purebred and had unsupervised access to the outside ***before*** installation of the ProtectaPet® system had lower scores, i.e., better welfare on this metric.

Regarding the Positivity sub-score, the MAM indicated that cats inherited from a previous owner and who were spending longer times outside ***after***the installation of ProtectaPet® had higher scores (better welfare). Cats with older owners had lower scores on this metric, while not having unsupervised rather than unsupervised access to the outside ***before***installation of the system showed a similar tendency.

For the Maintenance behaviors sub-score, the MAM included nine variables with six being clearly significant. Living within substantial grounds (compared to a small garden), being intact, and not having unsupervised (rather than unsupervised) access to the outside ***before***installation were associated with an increase in this sub-score (i.e., better welfare). Cats with older owners, and having cats entering the garden either ***before***or ***after***installation scored lower on this metric. An increasing number of cats in the home also had a marginal significance on lowering this score.

For Fearfulness the MAM included five predictors with one (whether the cat had an ongoing health problem) having marginal significance. Spending longer amounts of time outside ***before***installation, living with a substantial (rather than small) garden and living with more cats in the household resulted in a lower sub-score; whereas not being purebred was associated with higher scores.

## Discussion

To our knowledge this study is the first to investigate the impact of installing a physical containment system to provide a controlled outside environment for pet cats. Overall, the installation was associated with positive changes in both owner and cat quality of life, as assessed using the four-dimensional cat welfare scale developed as a result of this work. Time spent outside after installation had a significant effect on positivity and, to a lesser extent, maintenance behaviors. The majority of respondents lived in an urban environment with a relatively small garden, had multiple cats and a history of feline trauma associated with an RTA.

Although our sample was a self-selecting demographic based on those who had purchased some form of containment system, it is compositionally similar to that reported in other recent, larger UK-based surveys of cat owners, e.g., (27) (a random selected sample); (18), in terms of the proportion of cats who were neutered (>90%), the tendency for them to have restricted outside access (~45% vs. ~25% with unrestricted access). However, our data set had about twice the proportion of cats (35.6% vs. 17.5%) classified as purebred and half the proportion of cats with significant health problems (11.7 vs. 23.4%, respectively) compared to that reported by Finka et al. (18). Our data indicated that pedigree status was associated with increased health issues on our welfare subscale, thus it seems overall the cats in our sample were generally healthier. Approximately 20% of respondents were male, and this figure is similar to the proportion of cat owners involved in an earlier survey of UK cat and dog ownership using a randomized sampling method (28), but more than twice that reported by Finka et al. (18). Likewise, it seems the age of respondents is similar to that reported by Murray et al. (27), which is a slightly older profile than that described by Finka et al. (18). Overall, despite our survey focusing on those who had purchased a containment system, it seems the demographic of respondents appears to be quite similar to the wider UK cat-owning population, indicating that these products are not purchased by any particular type of cat owner.

Owners clearly believed in the importance of outdoor access with 77% agreeing or strongly agreeing with the statement “A cat that has access to the outdoors has a better quality of life than one that does not,” this is despite the majority of respondents having had a cat involved in a RTA, which was often fatal and this being a primary concern before installation of the system (85% of owners were very or strongly concerned about their cats being injured on the road). This strong belief in the importance of outside access for a healthy life among cat carers has been reported elsewhere (15) and seems, from the relationships seen in our data, to be strongly rooted in a belief that cats should have the freedom to express their natural behavior, the increased opportunities for exercise provided by outside access and for enrichment purposes, despite the obvious risks from traffic. It is therefore not surprising that the reliable safety provided by the system was the most important factor determining owners' decision to invest in it. Notable among the behaviors which were reported to change as a result of installation of the system were a reduction in soiling in the home, anxiousness and night time disturbance by a third of households, as well as a reduction in unexplained changes in mood and irritability by about a quarter and an increase in active relaxed behavior by about a quarter of households. Overall, these changes suggest general improvements in the cats' psychological well-being in many homes, and this is reinforced by the significant positive relationship between the positivity subscale and time spent outside.

Despite the emphasis apparently given to the importance of natural behavior patterns, freedom to hunt was seen as less important to cats and about a quarter of owners were very or extremely concerned about their cats killing wildlife. Just over 15% of owners considering wandering cats to be a nuisance, but around two thirds thought that owners should take responsibility for any problems caused to neighbors by their cat and nearly half thought that owners had a responsibility to stop their cat from wandering. Obviously, installation of the system not only reduces concerns about injury or loss of the cat, but also addresses concerns about the impact of their own cat on the neighborhood. Installation had little effect on hunting overall, with about 15% reporting an increase and a similar proportion a decrease, with about a third seeing no change and the rest reporting that it did not occur. We suggest, the variability in the impact of the system on hunting probably reflects differences in the local availability and distribution of prey both before and after the system has been installed in different locations. Another option would be that owners did not use to notice their cats hunting if they did not bring their prey to their home before the installation, suggesting that this behavior was reduced.

The vast majority of cats increased their time outside after installation of the system, with the amount of time typically doubling or more. There was no effect of the type of system installed on the amount of time spent outside, but this was associated with greater opportunity for those with the larger systems; not surprisingly, those with the more extensive systems, i.e., that covered most of the garden, saw a greater reduction in other cats entering the garden. About a third of those who suffered from bites and scratches, reported a reduction after installation. The only significant difference between the systems in changes in level of concern about the potential harm to their cat from being outside was related to getting lost where those with a “Cat fence barrier” were more concerned than those with a “Cat enclosure.” The difference between these two systems is that the former is installed over a pre-existent fence, unlike the latter. No further differences were found, including on the impact of their cat on wildlife.

The measures of well-being used in this study, grouped largely into four factors, two related to positive well-being and two related to negative well-being. While components of the same welfare valence were related it is worth noting that none of the constituent items cross loaded. Within the health issues subscale, there were not only signs of physical illness such as bites, breathing problems, cystitis and skin allergies, but also two behavioral items: house-soiling and unexplained changes in mood. The latter can be indicative of chronic pain/discomfort (29) and an association between house-soiling and cystitis is well-established (30, 31), which has been linked to environmental stressors (32) as has the risk of respiratory disease in certain contexts (33). A relationship between atopic skin disease and stress has also been postulated in cats (3) and recently shown in dogs (34) and it seems reasonable to suggest a similar mechanism may result in pruritus in cats. Thus, the subscale “Health issues,” appears to gather together a range of conditions associated with chronic stress with a risk of physical injury, but without an increase in aggressivity (which was associated with the Fearfulness factor). In this regard it is worth noting that in humans, chronic stress has been associated with impaired attention and executive function (35) and it might be that a similar phenomenon occurs in cats. Such a process would explain this association and highlights a potential role of chronic stress on the risk of road traffic accidents in cats, a major cause of fatality in this species (11), in addition to the free access to a road. Cats with unsupervised access to the outside before installation were more likely to show benefits in terms of their health scores. This suggests that unsupervised access had an impact to the animal's health and therefore these animals benefited from the security of the installation. Cats without unsupervised access to outside or indoors obviously did not benefit in the same way. Cats living in an urban environment (rather than semi-rural one) were at increased risk of health issues, which might also be related to stress from higher densities of unfamiliar cats. Thus, it may not be that being out *per se* is necessarily good for a cat, and more attention needs to be given to what might happen when out, given the local environment. The containment systems investigated here, provide an opportunity for safe exploration and increased control and these may be important features underpinning their benefit.

The other component related to poor welfare linked irritability and aggressive behavior with various indications of anxiety and fear in cats, including a tendency to hide away as well as clinginess (including excessive meowing). The relationship between behavior and emotional state has been identified previously for aggressivity toward both humans (36) and other cats (37). Interestingly (and perhaps contrary to expectations), a larger number of cats in the home was associated with a reduced risk of irritability/ aggressive behavior, but it should be noted that the target of aggression was not specified in the current study and previous studies have indicated that owner directed aggressive behavior is less common in multicat households (38). In addition, factor scores were also lower in those with substantial rather than small gardens, which would allow greater opportunity to avoid conflict. Lower levels of time outside before installation was associated with decreased fearfulness score, and this too would reinforce our earlier suggestion that being outside can be stressful for cats; by contrast, time spent outside after installation did not show any effect, suggesting that the security provided by the system was more able to alleviate the fears only for cats that was spending less time outside before the installation.

Playfulness is widely seen as a measure of positive well-being (39, 40) and so it is not surprising that willingness to play with the owner was related to being relaxed but active in their presence within the factor “Positivity.” Cats inherited from previous owners rather than obtained from other sources and those that spent more time outside after the containment system had higher positivity scores. This is again consistent with the suggestion that provision of a secure environment for cats may be key [they feel comfortable to explore the environment and gather information, i.e., the exploratory system is activated when the individual feels safe – (41)], and likewise a lack of play or relaxed activity around the owner might reflect a less secure environment.

The other subscale associated with good welfare linked a range of pleasurable activity (eating, drinking hunting and sleeping). Reductions in the first two of these have been associated with chronic stress and environmental predictability (32, 42) while sleep has been shown to be reduced in cats when they are kept in a stressful environment [quarantine cattery – (43)]. Thus, reductions in this subscale may reflect some sustained challenges to their well-being. Interestingly higher numbers of cats in the home had a tendency to reduce this score, despite also reducing fearfulness. This perhaps illustrates the danger of simple generalizations concerning the welfare significance of having a multicat households. However, the presence of cats from outside the home consistently lowered this score and can be considered a major threat to well-being. The risk of this was reduced by the more extensive containment systems and may be important if this is a problem. Owners with larger gardens and possibly whose cats spent more time outside after installation of the system, generally had cats with higher scores on this dimension, perhaps reflecting not only the opportunity for behavioral diversity but also engagement with it.

Limitations of this study include the inability to evaluate all of the potentially important factors that some might think could influence the outcomes assessed; such as how many owners were having their first cat; the socioeconomic profile of the owners; the current and age of cats when adopted. In addition, the study had a small sample size for some sub-groups (e.g., intact cats, cats inherited from previous owners), and so these results need to be treated with some caution. Also, there was no control group of cats included in the study; and owners had to recall whether their cats were showing less or more of the listed behaviors (i.e., it was not analyzed before and after the installation). Nonetheless it provides a useful first assessment and we would encourage a longitudinal study, with objective measures pre- and post-installation as a useful next step.

The physical barrier systems studied here appear to be highly effective in providing a controlled environment to the outside for cats without the risk inherent within some other systems e.g., electronic boundary systems (44). These systems appear to provide significant improvements to various aspects of cat welfare. The welfare subscales developed as part of this work provide a basis for more specific assessment of cat well-being, covering important but distinct components of welfare (stress related health issues, fearfulness, positivity and normal time-budgeting). These components are not entirely independent and so may be affected by changes in management in related and possibly opposing ways, reflecting more truly the complexity of the process of welfare assessment. The factors affecting subscale scores revealed here provide important insight into the intricate influences of the environment on well-being, and emphasis the danger of simple generalizations about this, beyond the importance of ensuring that the environment is safe and secure in every sense.

## Data Availability Statement

The datasets generated for this study are available on https://mfr.de-1.osf.io/render?url=https://osf.io/n5zx7/?direct%26mode=render%26action=download%26mode=render.

## Ethics Statement

The animal study was reviewed and approved by University of Lincoln Research Ethics Committee (ref: 2020-3442). Written informed consent was obtained from the owners for the participation of their animals in this study.

## Author Contributions

DM designed the questionnaire and collected the answers. LA and DM analyzed the data and wrote the manuscript text. All authors contributed to the article and approved the submitted version.

## Conflict of Interest

The research was funded by ProtectaPet^®^, producers of the systems analyzed in this study. However, its only involvement was sending the link to the questionnaire to its previous customers, thus, it was not involved with collection, analysis or interpretation of the data, nor with the writing of the manuscript. At no point did they have access to the raw data.
