# Supplementary material for: Introducing a Controlled Outdoor Environment Impacts Positively in Cat Welfare and Owner Concerns: The Use of a New Feline Welfare Assessment Tool
Source: Front Vet Sci. 2021 Jan 11;7:599284. doi: 10.3389/fvets.2020.599284 (PMC7829302; doi:10.3389/fvets.2020.599284)
Supplement: Supplementary file 1 [file Data_Sheet_1.docx]

Supplementary Material

# Caring for the cats survey

*Thank you for helping us with this survey, we are keen to promote healthier cat behaviour and lifestyles, and so are looking to gain greater insight into your experience with your ProtectaPet System. Your honest responses are very valuable to us. 
All information is gathered anonymously and will be collated by researchers at the University of Lincoln. In order to take part, we need you to confirm a few details about yourself (but nothing which will identify you), and then check that you understand that you will be giving us permission to use this information for our research. 
The data gathered here consists largely of responses to specific questions, although some of them are more open ended. We will not be able to identify who is responding to the survey and you can stop at any time, but it is understood that by completing the questions, you give us permission to use the information, including anonymous quotes in our work.
It is best if you complete this survey from a computer or tablet, rather than a mobile phone, as some of the tables may be difficult to see on a mobile phone. The survey should take no more than 10-15 minutes to complete.
Thank you for your attention.*Q2 Please confirm all of the following, we cannot use the information unless you are able to confirm all of the following:

▢ I am over 18 years old, and agree to give my honest opinion as far as possible (1)

▢ I have purchased a ProtectaPet fencing solution (2)

▢ I give you permission to use my data as outlined above (3)

Q3 Which Protectapet system have you purchased?

o Cat Fence Barrier (1)

o Cat Enclosure (2)

o Catio or Balcony (3)

Q4 Please tell us which gender you identify with?

o Male (1)

o Female (2)

o Other (3)

o Would rather not say (4)

Q5 How old are you?

o <18 years (1)

o 18-25 years (2)

o 26-35 years (3)

o 36-45 years (4)

o 46-55 years (5)

o 56-65 years (6)

o 66+ years (7)

Q6 What are your current personal circumstances?

o Healthy and able bodied (1)

o Registered disabled (2)

o Other ongoing significant health problems (3)

Q7 What country do you live in?

________________________________________________________________

Q8 Which of the following describes the area where you live?

o Urban (1)

o Rural (2)

o Semi-rural (3)

Q9 Which of the following best describes the land around your property?

o Yard/ small garden (8)

o family/medium sized garden (9)

o substantial garden/ private grounds (10)

Q10 Do you own more than one cat?

o Yes (1)

o No (2)

Skip To: Q13 If Do you own more than one cat? = No

Q11 How many cats do you own?

o 2 (1)

o 3 (2)

o 4 (3)

o 5+ (4)

Q12 If you have answered 'yes' to owning more than one cat, please answer all of the following questions with respect to your OLDEST cat, unless indicated otherwise

Q13 Where did you get your cat from?

o Shelter/rescue centre (1)

o Stray cat taken in (2)

o Purchased from pet shop (3)

o Purchased from breeder (4)

o Inherited from previous owner (5)

o Acquired from advertisement online/newspaper (6)

o Given by a friend/relative/acquaintance/private individual (7)

o Other, please specify (8) ________________________________________________

Q14 Is your cat purebred?

o Yes (1)

o No (2)

o Uncertain (3)

Q15 What gender is your cat?

o Male (1)

o Female (2)

o Not sure (3)

Q16 Has your cat been spayed/neutered?

o Yes (1)

o No (2)

o Not sure (3)

Q17 Has your cat been vaccinated in the last 12 months?

o Yes (1)

o No (2)

Q18 Is your cat microchipped?

o Yes (1)

o No (2)

Q19 Would you describe your cat as having an ongoing significant health problem?

o Yes (1)

o No (2)

o Uncertain (3)

Q20 Please indicate which of the following you think it is very important to provide to an indoor-only cat? (please select all that apply)

▢ Specific devices to encourage exercise (but not play) (1)

▢ Toys (2)

▢ Specific access to a sunny spot (3)

▢ Access to fresh air, e.g. slightly opened window (4)

▢ Food (5)

▢ Litter tray (6)

▢ Bed (7)

▢ Scratching post (8)

▢ Companionship (9)

▢ Vantage points / windows to look out from (10)

▢ Places to hide (11)

▢ Forms of enrichment not listed, please give brief details (12) ________________________________________________

Q21 BEFORE you purchased your ProtectaPet system, did your cat have intentional, unsupervised access to the outside, at least some of the time?

o Yes (1)

o No (2)

o I only got the cat after I installed the system (3)

Skip To: Q30 If BEFORE you purchased your ProtectaPet system, did your cat have intentional, unsupervised access... = I only got the cat after I installed the system

Q22 BEFORE you purchased your ProtectaPet system, how many hours per day did your cat typically spend outside?

o None (1)

o Less than 1 hour (2)

o 1 - 2 hours (3)

o 3 - 7 hours (4)

o More than 8 hours (5)

Q23 Please indicate if you have seen a change in any of the following in your cat since you purchased your ProtectaPet system

|  | condition has never occurred in my cats (1) | less severe/frequent now (2) | no change (3) | more severe/frequent now (4) |
| --- | --- | --- | --- | --- |
| Physical injuries - bites/ scratches (1) |  |  |  |  |
| Physical injuries - more serious (2) |  |  |  |  |
| Unexplained changes in mood (3) |  |  |  |  |
| Soiling in the home with either urine or faeces, including spray marking (4) |  |  |  |  |
| Cystitis / bladder problems diagnosed by the vet (5) |  |  |  |  |
| Respiratory/ breathing problems (6) |  |  |  |  |
| Skin allergies/ persistent scratching (7) |  |  |  |  |
| Hairballs (8) |  |  |  |  |
| Disturbance at night - crying / running around/ waking you up (9) |  |  |  |  |
| Willingness to play with you (10) |  |  |  |  |
| General presence around you (11) |  |  |  |  |
| Anxiousness/ jumpiness/ looking out the whole time (12) |  |  |  |  |
| Irritability/ aggressive behaviour (13) |  |  |  |  |
| Hunting behaviour / bringing prey home (14) |  |  |  |  |
| Thirst (15) |  |  |  |  |
| Appetite (16) |  |  |  |  |
| Sleeping (17) |  |  |  |  |
| Active but relaxed around the home (18) |  |  |  |  |
| Hiding away (19) |  |  |  |  |
| Clinginess, including excessive meowing (20) |  |  |  |  |
| Other, please specify (21) |  |  |  |  |

Q24 Does your cat now have greater access to the outside, compared to before you purchased the ProtectaPet system?

o Yes (1)

o No (2)

Q25 BEFORE you purchased your ProtectaPet system, which of the following were used to contain your cat when outside? (please select all that apply)

▢ I did not allow my cat outside (1)

▢ High fence/wall (2)

▢ Supervised access to the outside (3)

▢ Controlled access with electronic cat flap (4)

▢ Other commercial cat proof fencing (5)

▢ Cat enclosure/run (6)

▢ No method in place (i.e. unrestricted access to the outdoors) (7)

▢ Other (please specify) (8) ________________________________________________

Q26 Please indicate your level of concern regarding the following potential issues relating to your cat going outside, BEFORE you got the ProtectaPet system

|  | Unconcerned (1) | Slightly concerned (2) | Somewhat concerned (3) | Very concerned (4) | Extremely concerned (5) |
| --- | --- | --- | --- | --- | --- |
| Injury on the road (1) |  |  |  |  |  |
| Death on the road (2) |  |  |  |  |  |
| Getting lost (3) |  |  |  |  |  |
| Killing wildlife (4) |  |  |  |  |  |
| Conflict with other cats (5) |  |  |  |  |  |
| Conflict with other animals (6) |  |  |  |  |  |
| Problems for neighbours (7) |  |  |  |  |  |
| Poisoning (8) |  |  |  |  |  |
| Theft (9) |  |  |  |  |  |
| Getting trapped (10) |  |  |  |  |  |

Q27 BEFORE you purchased your ProtectaPet system, did any of the following issues influence the level of access to the outside that you gave your cat? (Please select all that apply)

▢ Risk of death or injury on the road (1)

▢ Disease risk from other cats (2)

▢ Getting lost (3)

▢ Killing wildlife/ leaving dead prey lying around (4)

▢ Conflict with other cats (5)

▢ Conflict with other animals (6)

▢ Problems for neighbours (7)

▢ Risk of poisoning from others (8)

▢ Theft (9)

▢ Getting trapped (10)

Q28 BEFORE you purchased your ProtectaPet system, how often do you think cats you do not own entered your garden?

o Less than once a month (1)

o Once a month (2)

o Once a week (3)

o More than once a week (4)

o once a day (5)

o More than once a day (6)

o Never (7)

Q29 How many hours per day does your cat typically spend outside now?

o None (1)

o Less than 1 hour (2)

o 1 - 2 hours (3)

o 3 - 7 hours (4)

o More than 8 hours (5)

Q30 Please indicate your level of concern regarding the following issues relating to your cat going outside, now, AFTER you have got the ProtectaPet system

|  | Unconcerned (1) | Slightly concerned (2) | Somewhat concerned (3) | Very concerned (4) | Extremely concerned (5) |
| --- | --- | --- | --- | --- | --- |
| Injury on the road (1) |  |  |  |  |  |
| Death on the road (2) |  |  |  |  |  |
| Getting lost (3) |  |  |  |  |  |
| Killing wildlife (4) |  |  |  |  |  |
| Conflict with other cats (5) |  |  |  |  |  |
| Conflict with other animals (6) |  |  |  |  |  |
| Problems for neighbours (7) |  |  |  |  |  |
| Poisoning (8) |  |  |  |  |  |
| Theft (9) |  |  |  |  |  |
| Getting trapped (10) |  |  |  |  |  |

Q31 Now that you have purchased a ProtectaPet system, how often do you think cats you do not own entered your garden?

o Less than once a month (1)

o Once a month (2)

o Once a week (3)

o More than once a week (4)

o once a day (5)

o More than once a day (6)

o Never (7)

Q32 Considering the following factors:

Please select the TWO MOST IMPORTANT factors that influenced your selection of the ProtectPet system that you chose

▢ Cost (1)

▢ Amount of space provided for the cat (2)

▢ Appearance (3)

▢ Ease of installation (4)

▢ Reliability / reputation for reliability (5)

▢ Safety provided by the system (6)

▢ Restriction of access to my garden by other cats (7)

▢ Other, please give details below (8)

Q33 Consider the following factors:

Please select the TWO LEAST IMPORTANT factors that would influence your selection of a containment method

▢ Cost (1)

▢ Amount of space provided for the cat (2)

▢ Appearance (3)

▢ Ease of installation (4)

▢ Reliability / reputation for reliability (5)

▢ Safety provided by the system (6)

▢ Restriction of access to my garden by other cats (7)

Q34 Please rate your level of concern regarding the following issues for cats in general that go on to other people's properties.

|  | Unconcerned (1) | Slightly concerned (2) | Somewhat concerned (3) | Very concerned (4) | Extremely concerned (5) |
| --- | --- | --- | --- | --- | --- |
| Conflicts with other cats (1) |  |  |  |  |  |
| Conflicts with other animals (2) |  |  |  |  |  |
| Disease risks (3) |  |  |  |  |  |
| Soiling of other people's property (4) |  |  |  |  |  |
| Spraying urine (5) |  |  |  |  |  |
| Coming into conflict with humans (6) |  |  |  |  |  |
| Destruction of the property (7) |  |  |  |  |  |
| Killing wildlife/ leaving dead prey items lying around (8) |  |  |  |  |  |
| Making lots of noise (9) |  |  |  |  |  |

Q35 Please rate the potential value you believe it is to a cat to have the following opportunities from going outside

|  | Not a benefit (1) | Slightly beneficial (2) | Somewhat beneficial (3) | Very beneficial (4) | Extremely beneficial (5) |
| --- | --- | --- | --- | --- | --- |
| Exercise (1) |  |  |  |  |  |
| Opportunities for hunting (2) |  |  |  |  |  |
| Increased space to wander explore / general enrichment (3) |  |  |  |  |  |
| Increased freedom to express natural behaviour (4) |  |  |  |  |  |

Q36 Have you ever had a cat injured on the road?

o Yes (1)

o No (2)

Skip To: Q38 If Have you ever had a cat injured on the road? = No

Q37 If you answered yes, was it fatal?

o Yes (1)

o No (2)

Q38 Please indicate how strongly you feel regarding the following statements

|  | Disagree strongly (1) | Disagree (2) | Neither agree nor disagree (3) | Agree (4) | Strongly agree (5) |
| --- | --- | --- | --- | --- | --- |
| A cat that has access to the outdoors has a better quality of life than one that does not. (1) |  |  |  |  |  |
| Cat owners should all have a specific method for containing their cats (2) |  |  |  |  |  |
| It is the owners responsibility to keep their cat from wandering (3) |  |  |  |  |  |
| Owners are responsible for any problems their cat can cause their neighbours (4) |  |  |  |  |  |
| The government should implement curfews for cats (5) |  |  |  |  |  |
| Wandering cats are a nuisance (6) |  |  |  |  |  |

Q39 Please indicate in a few words the single most valuable benefit to you or your cat from having the ProtectaPet system, please make sure you tell us why this is important.

________________________________________________________________

________________________________________________________________

**Supplementary Table 1 Descriptive analysis of the four principal components resulted from 21 behaviours and their improvement or not after installing one of the ProtectaPet systems. Trimmed mean 50% is calculated by discarding 25% of the lowest and the highest scores and then computing the mean of the remaining scores (i.e. it minimises the effects of extreme scores but is less conservative than median).**

| Principal Component | Interpretation | N | Mean | SD | Median | Trimmed Mean 50% | Min | Max | SE |
| --- | --- | --- | --- | --- | --- | --- | --- | --- | --- |
| 1 | Health issues | 248 | -0.09 | 0.16 | 0 | -0.08 | -0.85 | 0.28 | 0.01 |
| 2 | Positivity | 367 | 0.12 | 0.38 | 0 | 0.09 | -1 | 1 | 0.02 |
| 3 | Maintenance behaviours | 363 | 0.03 | 0.23 | 0 | 0.02 | -1 | 1 | 0.01 |
| 4 | Fearfulness | 339 | -0.07 | 0.26 | 0 | -0.05 | -1 | 0.71 | 0.01 |
